# Diffusive Electronic Transport Coefficients of Graphene/Bi_2_Se_3_ Heterostructures: Insights from First-Principles Calculations

**DOI:** 10.1021/acsomega.6c00680

**Published:** 2026-05-29

**Authors:** Alejandra J. de la Rosa-Jasso, Ricardo Alessandro Acosta-Martínez, Juan Hernández-Tecorralco, Lilia Meza-Montes

**Affiliations:** † 27779 Unidad Académica de Ciencia y Tecnología de la Luz y la Materia, Benemérita Universidad Autónoma de Zacatecas, Circuito Marie Curie S/N, Parque de Ciencia y Tecnología QUANTUM Ciudad del Conocimiento, Zacatecas 98160, Zacatecas, México; ‡ 211699 Instituto de Física, Benemérita Universidad Autónoma de Puebla, Puebla C.P. 72570, Puebla, México; § 87792 Instituto de Física, Universidad Nacional Autónoma de México, Ciudad de México C.P. 04510, México

## Abstract

Two-dimensional (2D) materials have emerged as versatile platforms for exploring novel physical phenomena, particularly in electronic and thermoelectric transport, where reduced dimensionality enables confinement effects that provide enhanced control over fundamental properties. In this context, van der Waals heterostructures offer a strategic route to engineering these properties via proximity effects. In this work, we investigate Graphene (G)/Bi_2_Se_3_ heterostructures, experimentally accessible monolayers, by means of density functional theory (DFT) calculations, including spin–orbit coupling (SOC) and van der Waals corrections. Two stacking configurations, *eclipsed* and *staggered* arrangements, are considered, both with favorable interlayer binding energies that confirm the stability of the interface. Our results, globally similar for both configurations, show that the physical response is dominated by interfacial coupling, which induces significant charge transfer resulting from the competition between the work functions of the individual components and the formation of an interface dipole. This charge transfer results in a metallic character in the heterostructures with an upward Dirac cone shift. Furthermore, SOC and band hybridization increase the complexity of the electronic structure, leading to features such as band splittings and avoided crossings due to proximity interactions. These combined effects give rise to an enhancement of both electrical and electronic thermal conductivities; however, they reduce the Seebeck coefficient and the electronic figure of merit ZT^(*e*)^, revealing a thermoelectric trade-off. Overall, we find that these heterostructures are better suited for efficient charge transport and heat dissipation than for thermoelectric energy conversion, underscoring how proximity effects can be exploited to tailor the functional limits of 2D systems.

## Graphical abstract

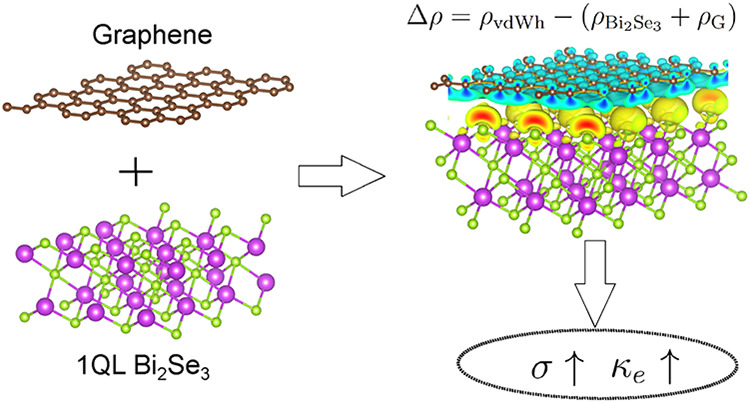

## Introduction

Two-dimensional (2D) materials have emerged as platforms for exploring a range of novel physical phenomena, for example those related to thermoelectric characteristics.^1–3^ Moreover, the current paradigm involves the assembly of materials by stacking different monolayers, i.e., van der Waals heterostructures (vdWh).^4^ The aim is to adjust their properties by integrating the distinct individual attributes into a new structure. G, the first isolated layer obtained experimentally,^5^ has a honeycomb crystal lattice and is one of the common choices to perform these studies due to its compliant integration.^6^ Its electronic bands exhibit a linear dispersion relationship, forming the well-known Dirac cones centered at the corners of its hexagonal Brillouin zone (BZ). Being a semimetal with excellent electrical conductivity, G is a good conducting material.^7,8^ However, graphene exhibits high electron mobility (200,000 cm^2^ V^–1^ s^–1^),^9^ which also results in high thermal conductivity, which consequently affects the figure of merit (ZT), making graphene a poor thermoelectric material.^10^

In contrast, layered materials such as Bi_2_Se_3_, Bi_2_Te_3_, and Sb_2_Te_3_ have demonstrated outstanding thermoelectric properties.^11^ These compounds belong to a class of quasi-two-dimensional quantum materials known as topological insulators (TIs).^12^ In particular, Bi_2_Se_3_ has a crystalline structure consisting of quintuple layers (QLs) with a Se–Bi–Se–Bi–Se sequence arranged in a *R* 3 *m* space group, and held together by weak van der Waals forces, which allow their exfoliation similar to graphene. Reports indicate the successful exfoliation of single QL or few QLs samples using electrochemical exfoliation on Bi_2_Se_3_ and Bi_2_Te_3_ crystals.^13^ The experimental bulk band gap of Bi_2_Se_3_ ranges from 0.2 to 0.3 eV^12,14^ and the theoretical predictions match very well to 0.3 eV.^15^ On the other hand, the conductivity properties of Bi_2_Se_3_ exhibit values of around 1.877 S/cm in an experimental study conducted by Patil et al.^16^ on thin films. Theoretically, the highest electrical conductivity (σ/τ) is obtained when the chemical potential is shifted within the range of −1.2 to −0.6 eV relative to the Fermi level. This corresponds to the *p*-type doping region where the density of states of Bi_2_Se_3_ becomes larger, as indicated by the ratio of electronic conductivity and relaxation time σ/τ ∼ 2000 × 10^17^ S/cm·s. However, the Seebeck coefficient is significantly weak in these regions, which is undesirable.^17^ As a topological material, Bi_2_Se_3_ has a band gap in its bulk while hosting metallic states on its surface. Their topological surface states arise due to time-reversal and spatial inversion symmetry along with strong SOC,^18^ and have been experimentally confirmed through ARPES measurements.^19^ The bulk properties of Bi_2_Se_3_ change when exfoliated to a specific number of QLs. The 1QL exhibits *P* 3 *m*1 symmetry and displays semiconducting behavior and lacks topological features. First-principles calculations for a 1QL predict an indirect bandgap of 0.89 (0.48) eV without (with) SOC. As the number of QLs increases, the band gap tends to close, as reported in refs 20,21 . Recent first-principles calculations coupled with model Hamiltonians by Zollner and Fabian reveal that the electronic bands of heterostructures formed by a monolayer or bilayer G in one, two, and three QLs of Bi_2_Se_3_ exhibit a band offset.^22^ This offset arises because of charge transfer from the G to the QLs as a result of the proximity interaction between layers. Similar results are reported in ref 23. Heterostructures composed of different materials have been successfully fabricated using a variety of experimental techniques, including molecular beam epitaxy (MBE), chemical vapor deposition (CVD), vapor-phase epitaxy, and sputtering.^24^ Graphene, experimentally realized in both monolayer and multilayer configurations,^25^ has been integrated with different monolayers such as transition metal dichalcogenides (TMDs), including MoS_2_ and WSe_2_, as well as hexagonal boron nitride (h-BN) to form vertically stacked heterostructures.^26,27^ Likewise, high-quality thin films of Bi_2_Se_3_ have been successfully grown using molecular beam epitaxy (MBE).^28^ Since both graphene and Bi_2_Se_3_ can be experimentally accessible as isolated systems, the fabrication of heterostructures based on these materials is feasible. In particular, heterostructures consisting of graphene and multilayer Bi_2_Se_3_ have been experimentally fabricated with well-defined and stable interfaces.^29^ In these systems, interfacial charge transfer, band alignment, and electronic effects were experimentally observed, leading to enhanced optoelectronic properties for different Bi_2_Se_3_ thicknesses, specifically for 5, 10, and 20 QLs.^29,30^ These results demonstrate the feasibility of forming stable heterostructures with graphene and Bi_2_Se_3_.

Despite these advances, the stack dependence on the formation energy and a systematic study of the thermoelectric properties remain lacking for heterostructures composed of G and the 2D limit of Bi_2_Se_3_ (1QL). For our study, we select this 1QL of Bi_2_Se_3_ as the representative material. While other possible topological insulators like Bi_2_Te_3_ are also prominent, Bi_2_Se_3_ is chosen due to its high structural stability with graphene and because its thermoelectric potential in this limit remains less explored in the literature. Therefore, the present work aims to provide a thorough analysis of these properties. To address these aspects, we conducted first-principles calculations coupled with semiclassical Boltzmann theory methods to characterize their energetic, electronic, and thermoelectric phenomena. First, we analyze the energetic stability and charge density transfer due to the proximity effects in heterostructures composed of G on a 1QL of Bi_2_Se_3_. We consider two different stacking configurations, and for these we examined their electronic structure considering SOC and also performed atomic projections. From the analysis of the electronic structure, we identify the formation of gaps attributed to avoided crossing points in the band structure induced by proximity interactions. Finally, we individually analyze each system and the overall effect on electrical and electronic thermal conductivities in their heterostructures.

## Computational Methods

To investigate the total energies, charge densities, and electronic and thermoelectric properties of the systems studied, we carried out first-principles calculations based on DFT, incorporating relativistic effects through SOC, as implemented in the Quantum ESPRESSO software package.^31^ The exchange-correlation energy was treated using the Generalized Gradient Approximation (GGA) in the Perdew–Burke–Ernzerhof (PBE) formulation.^32^ Norm-conserving, fully relativistic pseudopotentials were employed from the PseudoDojo library.^33^ For all systems, we used a plane-wave energy cutoff of 100 Ry and a Monkhorst–Pack *k*-point mesh of 27 × 27 × 1. To take into account van der Waals forces we include a Grimme-D3 correction^34^ to our calculations. Additionally, to prevent a dipole–dipole interaction along the *z*-axis a dipole correction was applied.^35^ For our study, we examined two different van der Waals heterostructures formed by G on a 1QL of Bi_2_Se_3_ in two different stacking arrangements, which we denoted eclipsed and staggered. In eclipsed stacking, a carbon atom is vertically aligned above a selenium, while in the staggered configuration the carbon atoms lie in the interstitial spaces of Bi_2_Se_2_. These configurations are shown in Figure 1b,c. We considered a (√3 × √3)*R*30° G supercell with *a*_G_ = 2.46 Å (the experimental lattice parameter of pristine G). This choice ensured a lattice mismatch of less than 5% when forming heterostructures with a Bi_2_Se_3_ unit cell. The lattice constant of Bi_2_Se_3_ is *a*_Bi_2_Se_3__ = 4.143 Å.^22^ The theoretical resulting lattice mismatch between G and Bi_2_Se_3_ was 1.17% after optimization. The G supercell contains 6 atoms, the 1QL system contains 5 atoms, as shown in Figure 1. Both heterostructures exhibit a lack of inversion symmetry. The optimized lattice constant of the heterostructures is *a* = 4.26 Å. The VESTA program was utilized for structural visualizations.^36^

**Figure 1:**
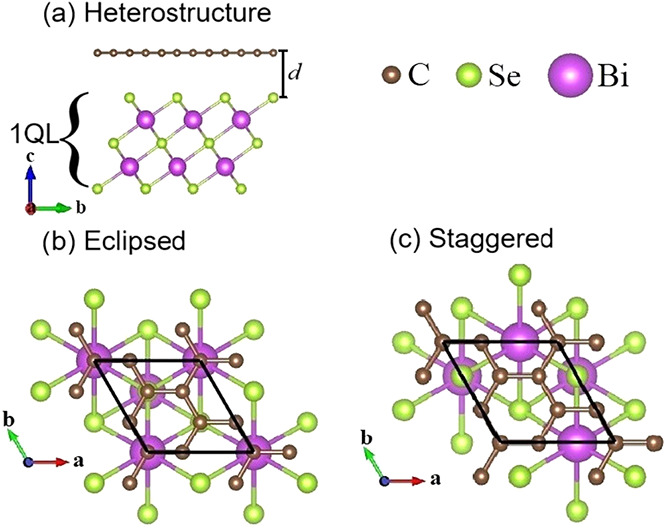
(a) Side view of the heterostructures formed by G on a 1QL of Bi_2_Se_3_. Top views of the stacking configurations: (b) eclipsed and (c) staggered.

To characterize the thermoelectric properties of the individual layers and their heterostructures, we constructed maximally localized Wannier functions (MLWFs) using Wannier90,^37^ based on *ab initio* results. As initial projections for the MLWFs, *s* and *p* orbitals were chosen for both Bi and Se while *p*_*z*_ orbitals for carbon since the electronic configurations indicate that the *d* orbitals do not play a significant role in these systems. Thermoelectric properties were evaluated with the BoltzWann module of Wannier90,^38^ which implements the semiclassical Boltzmann transport theory within the constant relaxation time approximation. The effect of SOC on the electronic behavior of Bi_2_Se_3_ is of significant relevance, as highlighted in ref 18. Hence, to accurately describe the electronic band structure, we incorporate the SOC contribution for all systems studied. In the Supporting Information, Figure S1 presents a comparison between the band structures obtained from DFT calculations and those reconstructed using Wannier interpolation. The excellent agreement observed validates the Wannier representation in the energy range relevant for the transport analysis. As our aim is to investigate the role of interfacial electronic coupling in charge and heat transport, we deliberately omitted the lattice thermal conductivity (κ_ph_) from our analysis. As we shall demonstrate, the electronic part of the figure of merit, ZT^(*e*)^, is itself low for heterostructures; including κ_ph_ would only further decrease the total ZT, hence omitting it does not change our main findings.

## Results and Discussion

### Energetic Stability and Electronic Properties of G/Bi_2_Se_3_ Heterostructures

To address the energetic stability of the two van der Waals heterostructures, with eclipsed and staggered stacking, we evaluated their interlayer binding energy *E*_b_ employing the following formula

1 $$E_{b}=\frac{E_{\mathrm{vdWh}}-\left(E_{{\mathrm{Bi}}_{2}{\mathrm{Se}}_{3}}+E_{G}\right)}{\mathrm{area}}$$

where *E*_vdWh_ is the total energy of the heterostructure, *E*_Bi_2_Se_3__ and *E*_G_ are the energies of Bi_2_Se_3_ and graphene,respectively, while *area* corresponds to the in-plane area of the supercell used in the construction of the heterostructure. The interlayer binding energy is shown in Table 1. For all cases, we observed favorable energies with magnitudes in the range of −10.75 to −11.69 meV/Å^2^. The heterostructure featuring staggered stacking exhibits a binding energy higher than that of its eclipsed partner, suggesting an enhanced energetic stability. To perform a thermodynamic analysis in relation to thermal fluctuations at room temperature (*k*_B_ *T* ∼ 25.7 meV at room temperature), we converted the interlayer binding energies into energy per unit cell by multiplying the density values times the *area* of the heterostructure. For the eclipsed configuration, the resulting energy is −168.88 meV per unit cell, whereas for the staggered configuration it is −183.65 meV per unit cell. Both values are significantly larger in magnitude than the thermal energy at room temperature, demonstrating that the G/Bi_2_Se_3_ heterostructure is thermodynamically stable against thermal fluctuations. Regarding stacking preference, the energy difference between the two configurations is 14.77 meV per unit cell. This suggests that, although the staggered configuration is energetically more favorable, thermal fluctuations at room temperature may facilitate the coexistence of multiple stacking arrangements within a single experimental sample. It is important to note that the magnitude of the interlayer binding energy corresponds to the exfoliation energy, providing a direct point of comparison with experimental measurements. Björkman et al. reported theoretical exfolation (binding) energies for several 2D materials such as graphene, hexagonal boron nitride (hBN), and MoS_2_ in the range of 13–21 meV/Å^2^,^39^ while experimental values for graphite’s exfoliation energy are typically reported around 28.7 meV/Å^2^.^40^ This energetic analysis indicates that these heterostructures are energetically favorable and stable under experimental conditions, supporting the feasibility of their experimental realization. The optimized lattice parameter for both stacking configurations is 4.26 Å. Upon relaxation, the interlayer separation, *d* (see Figure 1 and Table 1) observed between G and 1QL of Bi_2_Se_3_ measures *d* = 3.54 Å in the eclipsed stacking, and *d* = 3.48 Å in the staggered configuration. In the eclipsed case, the alignment of carbon atoms directly above the Se atoms induces interlayer repulsion due to overlapping of electronic clouds, resulting in an increased interlayer spacing and lower *E*_b_ compared to the staggered one. This analysis provides a theoretical basis for proposing that the staggered stacking arrangement should be more stable experimentally.

**Table 1:** Interlayer Binding Energy *E*_b_ and Interlayer Distance *d* of G/Bi_2_Se_3_ Heterostructures for Different Stacking Configurations

System	stacking	*E*_b_ (meV/Å^2^)	Interlayer distance *d* (Å)
G/Bi_2_Se_3_	eclipsed	–10.75	3.54
G/Bi_2_Se_3_	staggered	–11.69	3.48

To analyze the effect of the proximity of G to 2D Bi_2_Se_3_ layers on the electronic properties, we first calculate the charge density redistribution due to the heterostructure’s formation. This redistribution of charge density was determined by taking the difference between the charge density of the heterostructure, ρ_vdWh_, and the sum of the charge densities of the individual constituents, ρ_Bi_2_Se_3__ and ρ_G_

2 $$\Delta \rho =\rho_{\mathrm{vdWh}}-\left(\rho_{{\mathrm{Bi}}_{2}{\mathrm{Se}}_{3}}+\rho_{G}\right)$$

Figure 2 displays the charge density distribution for both top and lateral views of the heterostructures. The yellow isosurfaces denote regions of charge accumulation, while the cyan isosurfaces indicate areas where charge is depleted. In other words, there is a charge transfer from regions exhibiting cyan to yellow isosurfaces, i.e., from G to the 1QL of Bi_2_Se_3_. This effect is observed in both stacking arrangements, indicating that electron transfer proceeds from G to Se atoms. According to Figure 2b, within the staggered system, the transfer of charge is significantly more evident. Significant electron transfer is expected to energetically shift the electronic bands of individual layers during heterostructure formation.

**Figure 2:**
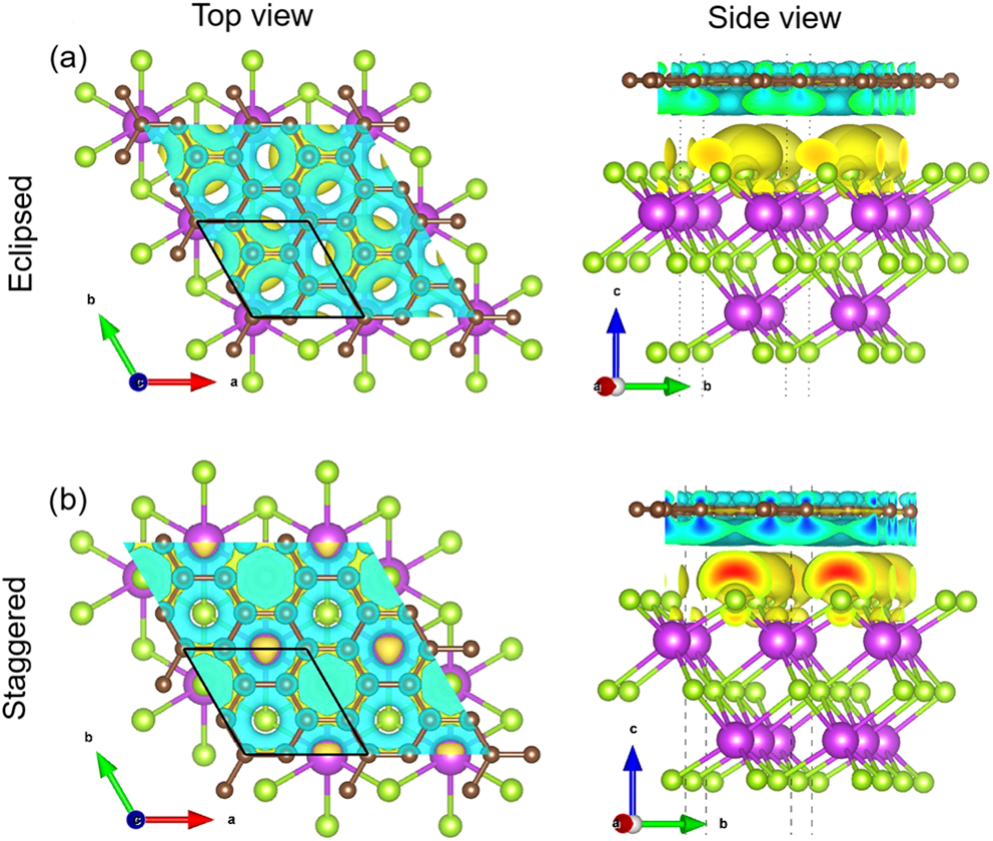
Charge redistribution, with SOC, for the eclipsed and staggered stackings of G/Bi_2_Se_3_. The isosurface is set to 2 × 10^–4^ e/Å.

As previously stated, we consider a G superlattice of (√3 × √3)*R*30° on top of a unit cell of Bi_2_Se_3_ in our heterostructures. This arrangement ensures that the layers remain commensurate. As a result, the electronic structure of G changes since its characteristic Dirac cone is folded to the Γ point within the supercell BZ (see Figure 3a). The SOC interaction in G is weak as a consequence of its low atomic mass,^41^ and therefore the Dirac cone is preserved. As expected, the use of supercells does not induce electronic changes, as reported in refs 8,10 , and corroborated by the density of states in Figure 3a which reflects the linear energy dispersion of graphene in the vicinity of the Dirac cone, as has been reported in previous studies. Figure 3b presents the electronic band structure and density of states of a single quintuple layer of Bi_2_Se_3_ including SOC. From this Figure, we identify an indirect band gap of 0.366 eV for 1QL of Bi_2_Se_3_. The valence band maximum is located away from high-symmetry points, while the conduction band minimum lies at the Γ point. Another distinctive feature is the asymmetric behavior of the DOS between the valence and conduction bands which is not present in graphene. This asymmetry leads to a higher Seebeck coefficient, as will be discussed in subsequent sections. The band structures of graphene and 1QL of Bi_2_Se_3_, with and without SOC, are presented in Figures S2 and S3 in ref 42. In the absence of SOC, the 1QL exhibits an indirect band gap of 0.799 eV. These results highlight the strong influence of SOC on the electronic structure of Bi_2_Se_3_, in agreement with previous theoretical studies.^22^

**Figure 3:**
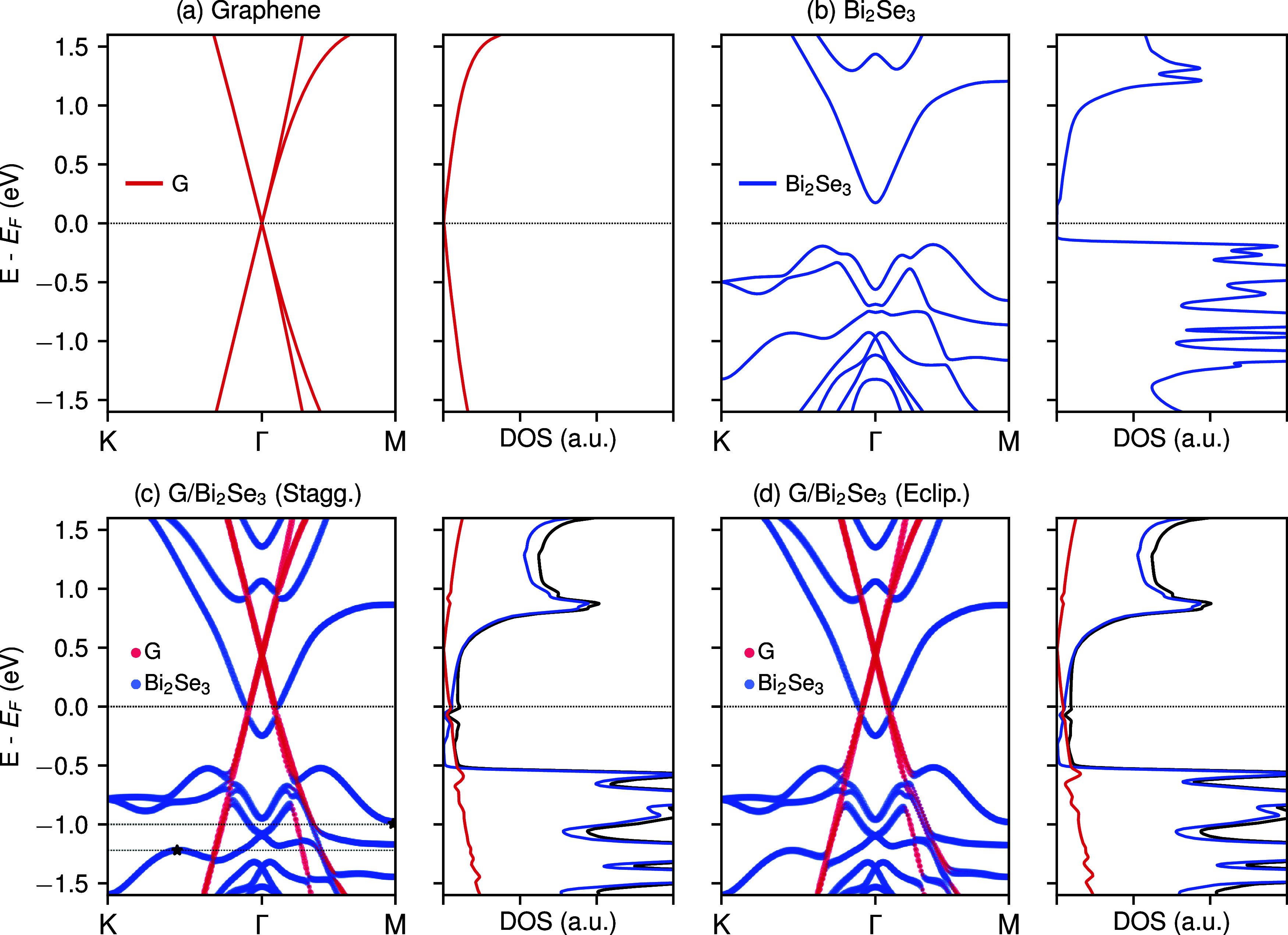
Electronic band structures and density of states for (a) Graphene, (b) 1QL of Bi_2_Se_3_, and the (c) eclipsed and (d) staggered G/Bi_2_Se_3_ heterostructures, both including SOC. Red and blue dots indicate contributions from G and Bi_2_Se_3_, respectively. The Fermi level is set at zero. The dotted line in (c) highlights two points (marked with star symbols in black) where the curvature changes. In (c, d), the color-coded bands represent the fat-band projection of the electronic states, where red and blue denote the relative contributions from the graphene and Bi_2_Se_3_ layers, respectively.

Figure 3c,d present the band structures and density of states for the two heterostructures, eclipsed and staggered. The color-coded bands represent the fat-band projection of the electronic states, where red and blue denote the relative contributions of graphene and Bi_2_Se_3_ layers, respectively. We restricted our analysis to the energy range from −1.5 to 1.5 eV around the Fermi level, since this region contains the states that dominate low-energy electronic behavior and transport properties. For both cases, a metallic character is observed, unlike the semimetallic nature of G and the semiconducting characteristics observed for isolated thin layers of Bi_2_Se_3_.^43^ The metallicity arises from charge transfer caused by the interfacial layer interaction, and the original bands of each constituent experience a relative energy shift, as mentioned above. The projected band structures clearly reveal the folded Dirac cone of G at Γ, depicted in red, as well as the valence and conduction bands of the 1QL unit cell of Bi_2_Se_3_ (blue lines).

Analysis of the band structures reveals a systematic shift of the Dirac cone approximately ∼0.42 eV above the Fermi level in both stacking configurations, indicating *p*-type doping of G induced by its interaction with the Bi_2_Se_3_ substrate. Consequently, the bottom of the conduction band of Bi_2_Se_3_ shifts below the Fermi level by about ∼0.2 eV. These observations are consistent with the charge-density difference maps previously discussed, which revealed electron transfer from G to Bi_2_Se_3_, and they are in good agreement with previous studies.^22^ For both heterostructures, the density of states (DOS) near the Fermi level exhibits a nearly uniform behavior. The band-shift effect can be understood quantitatively from the difference in work functions and the formation of an interfacial dipole (as illustrated in Supporting Figure S4). Our calculations yield Φ_G_ = 4.25 eV for isolated graphene and Φ_Bi_2_Se_3__ = 5.59 eV for 1QL of Bi_2_Se_3_. Since Φ_Bi_2_Se_3__ > Φ_G_, electrons transfer from graphene to Bi_2_Se_3_ upon contact, driving the observed *p*-type doping of graphene. According to the Schottky–Mott rule, the ideal Fermi level shift should equal the work function difference ΔΦ = Φ_Bi_2_Se_3__ – Φ_G_ = 1.34 eV. However, the formation of an interfacial dipole Δ*V*_dipole_ = 0.84 eV at the van der Waals interface (arising from charge redistribution and Pauli repulsion) screens part of this driving force. The condition for electrostatic potential conservation

3 $$\Delta \Phi =\Delta V_{\mathrm{dipole}}+\Delta E_{F}$$

requires a Fermi level shift of Δ*E*_F_ = 0.50 eV. Although the individual band shifts of each constituent are not strictly required to independently match the global Δ*E*_F_, as needed for electrostatic balance, the observed shift of the Dirac cone is 0.42 eV, which nearly reproduces Δ*E*_F_, with a tiny discrepancy of 0.08 eV. This difference could be attributable to atomic relaxation and local charge reorganization in the fully optimized heterostructure. Also, it is important to mention that the asymmetry between the shifts of the Dirac cone (0.42 eV) and the Bi_2_Se_3_ conduction band (0.20 eV) is a consequence of the different DOS of each material. The vanishing DOS of graphene near the Dirac point makes it more sensitive to charge transfer than the parabolic bands of Bi_2_Se_3_, causing larger energy shifts for the same amount of transferred charge.

Beyond these global features, a closer comparison between stacking configurations reveals subtle but meaningful differences. The staggered arrangement induces stronger interfacial hybridization between graphene π states and Se *p* orbitals, as evidenced by the more pronounced widening of the bands and DOS. This enhanced interaction leads to a more noticeable gap opening near *E*_F_, clearly visible in the DOS, which arises from the reduced interlayer separation in the staggered stacking. In contrast, the eclipsed stacking exhibits weaker coupling and, consequently, a less significant electronic perturbation.

Of course, the band structure of the heterostructure should reflect the interaction between the out-of-plane orbitals of G and the 1QL of Bi_2_Se_3_. Near the Fermi level, where the bottom of the Bi_2_Se_3_ conduction band now resides, avoided crossings or electronic discontinuities appear in the regions where the bands cross. Observation of avoided crossings in the band structure suggests a local hybridization between electronic states of different nature, resulting in energy splitting which is due to electronic repulsion between bands from distinct layers.^44^ The DOS shows a depletion below the Fermi level, confirming the band structure’s avoided crossing at these energies. In contrast to the band structure of graphene, that exhibits a folded pattern in Figure 3a, the band structure in Bi_2_Se_3_ corresponds to its primitive cell, indicating that the electronic repulsion observed is a result of its interaction with G. At energies below −0.5 eV, on the other hand, a significant hybridization of the Bi_2_Se_3_ and G bands is evident. This behavior indicates that an electron traveling on G could jump to Bi_2_Se_3_ and vice versa. At deeper energies, additional avoided crossings arise due to coupling among bands from different layers.

An unfolding analysis was conducted in the heterostructure with the staggered stacking to explore the evolution of the original graphene electronic states due to the interaction. First, we unfold the electronic band structure of each layer onto the primitive graphene lattice. For a direct comparison with the unfolded bands of the heterostructure, the graphene bands (red dots in Figure 4a) are adjusted by approximately ∼0.42 eV upward of the Fermi level, while the bottom of the Bi_2_Se_3_ conduction band is moves downward by ∼0.2 eV (black arrows in Figure 4a). Additionally, their valence bands are also shifted toward the Fermi level, indicating that the interaction between layers not only alters the electronic structure, but also tunes the band gap in Bi_2_Se_3_ (blue dots in Figure 4a). Two conduction bands of Bi_2_Se_3_ intersect the Fermi level at Γ and *K* points. The additional band crossing at the *K*-point arises from the mapping of its unit cell in the reciprocal space to the graphene-based representation. In contrast, the graphene unfolded bands show a characteristic and well-defined Dirac point at *K*, because the projection matches to its true periodicity. At the Γ point in the unfolded band structure of the heterostructure, Figure 4b, we observe the isolation of the bottom of the Bi_2_Se_3_ conduction band, just below the Fermi level, as shown in Figure 3. Around the *K*-point, the linear dispersion of the graphene bands remains in the vicinity of the Dirac point; however, between −0.5 eV and −1.0 eV, several electronic anticrossings appear as gaps along the Γ–K path, where they intersect with the bands of the 1QL of Bi_2_Se_3_. As is evident in Figure 4, an interesting aspect of the interlayer coupling is the change in the dispersion of the Bi_2_Se_3_ bands near the *K* point, indicating a strong hybridization between states of the separate layers.

**Figure 4:**
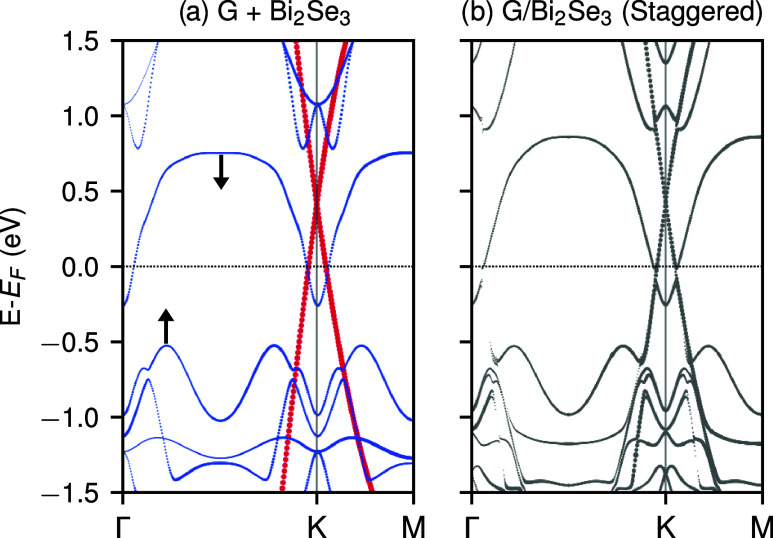
(a) Unfolded band structures for individual systems: For the G (red dots) and Bi_2_Se_3_ (blue dots) energy levels, shifts in energy were done as indicated by the black arrows, for comparison to the heterostructure bands. (b) Unfolded band structure of the system G/Bi_2_Se_3_ with staggered stacking. The Fermi level is set to zero.

In general, these results demonstrate that the interfacial interaction between graphene and Bi_2_Se_3_ not only alters the energy alignment of their electronic states but also induces emergent phenomena such as band splitting and band hybridization. Following the analysis of electronic properties, the next section offers a thorough examination of the thermoelectric properties of the individual components and their heterostructures, under identical computational parameters. The thermoelectric response was assessed across chemical potentials ranging from −1.5 to 1.5 eV to simulate varying doping levels, establishing a direct connection between the electronic band structure and the temperature-dependent transport coefficients.

### Proximity Effects on Electronic and Thermal Diffusive Transport in G/Bi_2_Se_3_ Heterostructures

Chae et al.^45^ experimentally investigated heterostructures composed of graphene and few layers of Bi_2_Se_3_. They identified three distinct regimes for the temperature-dependent resistance: a saturation below 4 K due to Se vacancies; a linear behavior dominated by electron–electron scattering as temperature increases up to some tens of K; and finally, an exponential growth due to lattice vibrations. Based on these observations, our study focuses on the diffusive regime. This choice is physically justified by the fact that operational devices typically contain defects, which limit the mean free path, and function at temperatures where scattering processes are dominant, as observed by Chae et al. By aligning our model with this experimental evidence, we ensure that our analysis reflects realistic conditions found in practical electronic and thermoelectric applications.

Although both bulk Bi_2_Se_3_ and Bi_2_Te_3_ are well-established thermoelectric materials due to their narrow band gaps and layered crystal structures, less attention has been paid to their 2D forms.^46^ In particular, the single QL of Bi_2_Se_3_ remains underexplored in terms of its thermoelectric properties, despite their distinct behavior due to dimensional confinement and reduced thermal conductivity.^47^ In contrast, due to its high thermal conductivity, G is considered a poor thermoelectric material.^10^ In general, confinement from the bulk to two dimensions is expected to enhance the power factor and reduce the phonon thermal conductivity.

Heterostructures of G on a 1QL of Bi_2_Se_3_ exhibit notable alterations in electronic properties due to interlayer interactions. The semimetallic properties of graphene and the semiconducting characteristics of Bi_2_Se_3_ are altered, leading to a metallic behavior. Therefore, a comprehensive analysis of the thermoelectric properties of these materials and their heterostructures is necessary to gain a deeper physical understanding. Thermoelectric features are computed using semiclassical Boltzmann transport theory, within the constant relaxation-time approximation (CRTA) for each component and for both heterostructure stacking arrangements. It is well-known that the CRTA has limitations, as scattering mechanisms may depend on several parameters such as energy, momentum, and carrier or impurity concentrations.^48^ In layered systems, specific mechanisms arise; for instance, graphene exhibits unique features like flexural phonon modes.^49^ Additional scattering mechanisms have been identified in heterostructures, such as interfacial scattering in G/MoS2 driven by phonon modes emerging at the interface of layered systems.^50^ Nevertheless, the CRTA has given results comparable to experiments, and is used to predict the behavior of systems of diverse dimensionality: the bulk^51^ (and references therein), the Bi_2_Se_3_ monolayer^52^ and few QLs of Bi_2_Se_3_.^53^

For calculation of electrical (σ) and thermal conductivities (κ_e_) we employ the following known values: for G, τ = 3 × 10^–13^ s^54^ and for 1QL Bi_2_Se_3_, τ = 1.52 × 10^–13^ s, a value obtained by applying the expression provided in.^55^ For the heterostructure, the mean τ = 2.26 × 10^–13^ s was taken, reflecting the joint contribution of both materials. Figure 5 shows the variation of the thermoelectric properties as a function of the chemical potential (μ), in the range of −1.5 to 1.5 eV for G, a 1QL of Bi_2_Se_3_, and their heterostructures in both staggered and eclipsed arrangements, at a temperature of 300 K. To bridge the theoretical calculations with experimental conditions, the chemical potential range was mapped to carrier densities. While the full interval serves as a computational probe to examine electronic trends in the high-density regime, the chemical potential at ±0.2 eV corresponds to carrier density of 10^13^ carriers/cm^2^, while at ±0.5 eV the density reaches 10^14^ carriers/cm^2^. These regimes are achievable through electrostatic gating based on ionic-liquid techniques.^56,57^ This approach ensures that our transport analysis covers both typical experimental conditions and the extreme doping limits reported in theoretical studies. Notice that μ < 0 corresponds to a hole-doped or *p*-type region, while μ > 0 refers to electron-doped or *n*-type behavior.

**Figure 5:**
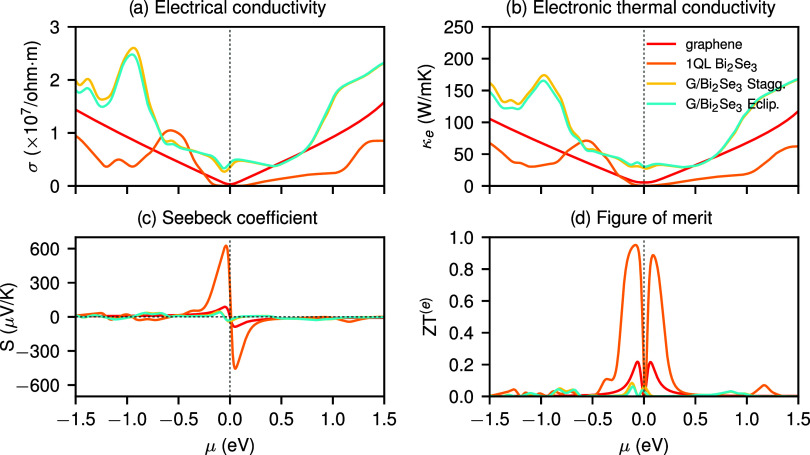
(a) Electrical conductivity (σ), (b) electronic thermal conductivity (κ_e_), (c) Seebeck coefficient (*S*), and (d) electronic component of figure of merit (ZT^(*e*)^) for G, single QL of Bi_2_Se_3_ and their heterostructures at *T* = 300 K.

The red lines in Figure 5a,b for graphene show a linear relationship in electrical and thermal conductivity as a function of μ, consistent with the semimetallic behavior and the linear-dispersion band around the Dirac point.^58^ In the case of a single QL of Bi_2_Se_3_ (orange lines in Figure 5a,b), both conductivities show an asymmetric behavior respect to μ = 0, in agreement with the indirect semiconducting behavior. Also, within the examined range of chemical potential in the band gap region, the material exhibits zero conductivity, while the maximum electrical conductivity occurs in the zone of *p*-type region at μ = −0.65 eV, where reaches a value greater than 1 × 10^7^ ohm^–1^ m^–1^. At μ = 1.4 eV, the Bi_2_Se_3_ shows a second conductivity maximum of approximately 0.8 × 10^7^ ohm^–1^ m^–1^.

According to the Wiedemann–Franz law, electronic thermal conductivity is directly proportional to electrical conductivity, therefore σ and κ_e_ are expected to have similar tendencies as demonstrated in Figure 5. The Seebeck response of the isolated layers reflects their intrinsic electronic properties. Given that graphene exhibits nearly symmetric π bands around the Fermi level, minimal electron–hole asymmetry results and, consequently, a low Seebeck coefficient; whereas the asymmetric density of states of Bi_2_Se_3_ near the band edges produces a stronger Seebeck effect. In the latter case, the Seebeck coefficient is within the range of 400 to 600 μV K^–1^, the value depending on the region, either *p*-type or *n*-type. Figure 5d shows the electronic component of the figure of merit, ZT^(*e*)^ = S^2^σ*T*/κ_e_, which represents the theoretical upper limit achievable in the absence of thermal conductivity of the lattice. Realistic ZT values shall be reduced when κ_ph_ is considered. In graphene, the figure of merit is relatively low, around 0.18, attributed to its low Seebeck coefficient and elevated κ_e_. In contrast, the single QL of Bi_2_Se_3_ exhibits higher ZT values, for μ = −0.26 eV where S is positive and large (*p*-type region), ZT^(*e*)^ = 0.98; and for μ = 0.2 eV, where *S* is negative (*n*-type), ZT^(*e*)^ = 0.89. This large value indicates its potential as a promising 2D thermoelectric material.

Since our main goal is to analyze the thermoelectric transport properties at the van der Waals interface between G and Bi_2_Se_3_, we now focus on their heterostructures, comparing both stacking configurations to the individual components. Our calculations reveal enhancement in both electrical conductivity and electronic thermal conductivity, exhibiting values greater than those of the individual components throughout the range of chemical potential examined, except near −0.5 and 0.5 eV, where Bi_2_Se_3_ and G display larger values, respectively. The enhancement in conductivities is slightly stronger in the staggered arrangement (golden lines in Figure 5) compared to the eclipsed configuration (cyan lines in Figure 5) in the *p*-type regime. Conversely, near μ = 0, this behavior reverses and becomes nearly unaffected by stacking in the *n*-type region. The increase in σ and κ_e_ confirms the charge transfer and band hybridization between the π states of G and the 1QL-Bi_2_Se_3_ states at the interface discussed above. The stacking dependence of these values indicates that the geometric alignment could influence the interlayer coupling and, consequently, the carrier mobility. Nevertheless, the formation of vdWhs decreases the Seebeck coefficient due to a more symmetrical density of states near the Fermi level. Despite the improvement in electrical transport, the overall thermoelectric efficiency decreases as a result of low S values, significantly reducing in turn the figure of merit of the heterostructures compared to both pristine G and 1QL Bi_2_Se_3_.

Figure 6a,b show the electrical and electronic thermal conductivities, respectively, of the staggered G/Bi_2_Se_3_ heterostructure as a function of the chemical potential at various temperatures. A linear scale is used for σ because it exhibits only small variations within the studied range of chemical potential, while κ_e_ is presented on a logarithmic scale to highlight its changes by orders of magnitude. As the temperature increases, the thermal smearing maintains a stable electrical conductivity in the heterostructure. Slight variations occur in the *p*-type region where σ changes with increasing temperature, while in the *n*-type region σ remains unaffected by the temperature. These alterations arise from the behavior of the electronic structure via the formation of the heterostructure; for example, the slight increase of σ with temperature just below the Fermi level could be attributed to the avoided crossings discussed above. Between −1.0 and −1.2 eV, fluctuations in σ could be correlated to bands with nearly zero group velocity but different concavity from the edges of deep valence bands of a single QL of Bi_2_Se_3_, as shown by a black line with a star symbol in Figure 3c. On the other hand, electronic thermal conductivity κ_e_ shows a clear temperature dependence, increasing and smoothing with temperature. This significant temperature dependence of κ_e_ can be understood by the term (ϵ – μ)^2^ in the integral of heat transport,^38^ which enhances the influence of electronic states away from the chemical potential. As temperature rises, thermal broadening allows high-energy states to contribute to heat conduction, which significantly increases κ_e_ and smooths the characteristic peaks observed at low temperatures.

**Figure 6:**
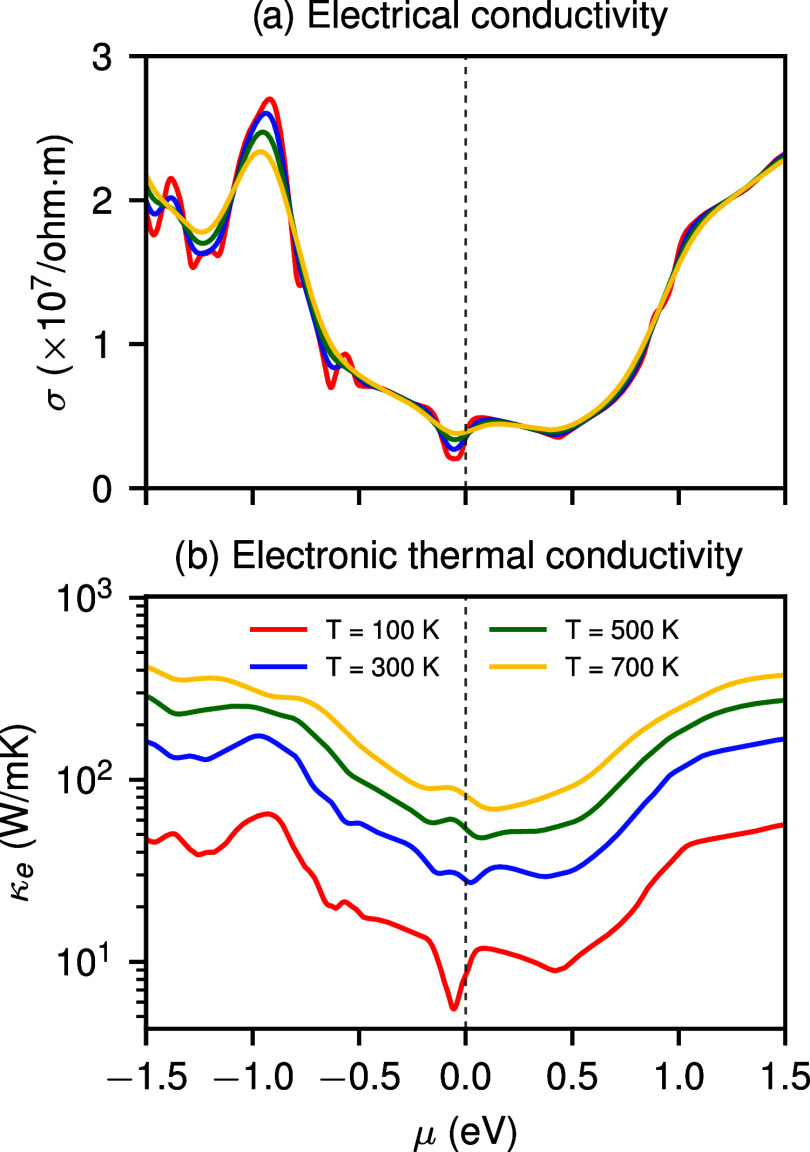
(a) Electrical conductivity (σ), and (b) electronic thermal conductivity (κ_e_) for the staggered G/Bi_2_Se_3_ heterostructure, as a function of chemical potential at various temperatures. A linear scale is employed for σ, while κ_e_ is presented on a logarithmic scale.

The behaviors of σ and κ_e_ indicate that the G/Bi_2_Se_3_ heterostructure enhances electronic transport relative to individual layers. However, this conduction enhancement fails to improve the thermoelectric efficiency because the simultaneous increase in κ_e_ and the reduction of the Seebeck coefficient decrease the figure of merit. The simultaneous enhancement of σ and κ_e_ highlights the potential of these heterostructures for nanoelectronic and optoelectronic applications that require high carrier mobility and efficient heat dissipation.

## Conclusions

In summary, this study provides a comprehensive first-principles analysis of G/Bi_2_Se_3_ heterostructures, demonstrating how interfacial proximity effects induce significant changes in the electronic and thermoelectric response compared to standalone components. We find favorable interlayer binding energies of −10 meV/Å^2^ which suggest that these interfaces are experimentally feasible in two different stacking arrangements, namely eclipsed and staggered. The interaction at the interface leads to a significant redistribution of charge, driven by the interplay between the work function mismatch and the formation of an interface dipole. This charge transfer, combined with band hybridization and spin–orbit coupling, drives the system into a metallic state characterized by a shifted Dirac cone and the emergence of avoided crossings in the band dispersion. Our results confirm that while the specific stacking introduces slight changes, the overall transport behavior remains qualitatively consistent. A key finding of this work is the identification of a fundamental thermoelectric trade-off. The interfacial mechanisms which induce changes in the electronic properties enhance electrical (σ) and electronic thermal (κ_e_) conductivities while simultaneously suppress the Seebeck coefficient (*S*) and the electronic figure of merit (ZT^(*e*)^). This trade-off is a direct consequence of metallic behavior and the reduced local asymmetry in the density of states around the Fermi level. This local symmetry significantly reduces the efficiency with which carriers are energy-filtered. Our findings indicate that, despite challenges for thermoelectric conversion, the simultaneous rises in both electrical and electronic thermal conductivities could be beneficial for applications that require efficient charge transfer and heat dissipation.

## Supplementary Material



## Notes

Comparison between band structures obtained from DFT and Wannier interpolation for graphene, Bi_2_Se_3_, and the heterostructures in staggered and eclipsed configurations (Figure S1); the electronic band structure of pristine graphene and their band-folded structure in the (√3 × √3)*R*30° supercell (Figure S2); the effect of spin–orbit coupling in the band structure of a 1QL of Bi_2_Se_3_ (Figure S3); the planar-averaged electrostatic potential and energy level alignment, including the work function and interfacial dipole in the staggered heterostructure (Figure S4); the transport coefficients, including electrical and electronic thermal conductivities as a function of the chemical potential for graphene, a 1QL of Bi_2_Se_3_, and G/Bi_2_Se_3_ heterostructures in staggered and eclipsed configurations (Figure S5); and the Seebeck coefficient of graphene at different temperatures (Figure S6), and lists representative room-temperature Seebeck coefficients reported for single-layer graphene on different substrates, included here as reference values for comparison with our results (Table S1), and Figures S5 and S6 are mainly included to provide additional visualization of the transport behavior and to support the trends discussed in the main text (PDF)
