# Supplementary material for: Diffusive Electronic Transport Coefficients of Graphene/Bi2Se3 Heterostructures: Insights from First-Principles Calculations
Source: ACS Omega. 2026 May 29;11(23):33920–9. doi: 10.1021/acsomega.6c00680 (PMC13281020; doi:10.1021/acsomega.6c00680)
Supplement: Supplementary file 1 [file ao6c00680_si_001.pdf]

Diffusive Electronic Transport Coefficients of Graphene/Bi_2Se_3 Heterostructures: Insights from First-Principles Calculations

Alejandra J. de la Rosa-Jasso,[†,‡] Ricardo Alessandro Acosta-Martínez,[‡] Juan Hernández-Tecorralco,[*,¶] and Lilia Meza-Montes[*,‡]

†*Unidad Académica de Ciencia y Tecnología de la Luz y la Materia, Benemérita Universidad Autónoma de Zacatecas, Circuito Marie Curie S/N, Parque de Ciencia y Tecnología QUANTUM Ciudad del Conocimiento, 98160 Zacatecas, Zacatecas, México.*

‡*Instituto de Física, Benemérita Universidad Autónoma de Puebla, Puebla C.P. 72570, Puebla, México.*

¶*Instituto de Física, Universidad Nacional Autónoma de México, Ciudad de México, C.P. 04510, México*

E-mail: juanht@fisica.unam.mx; lilia.meza@correo.buap.mx

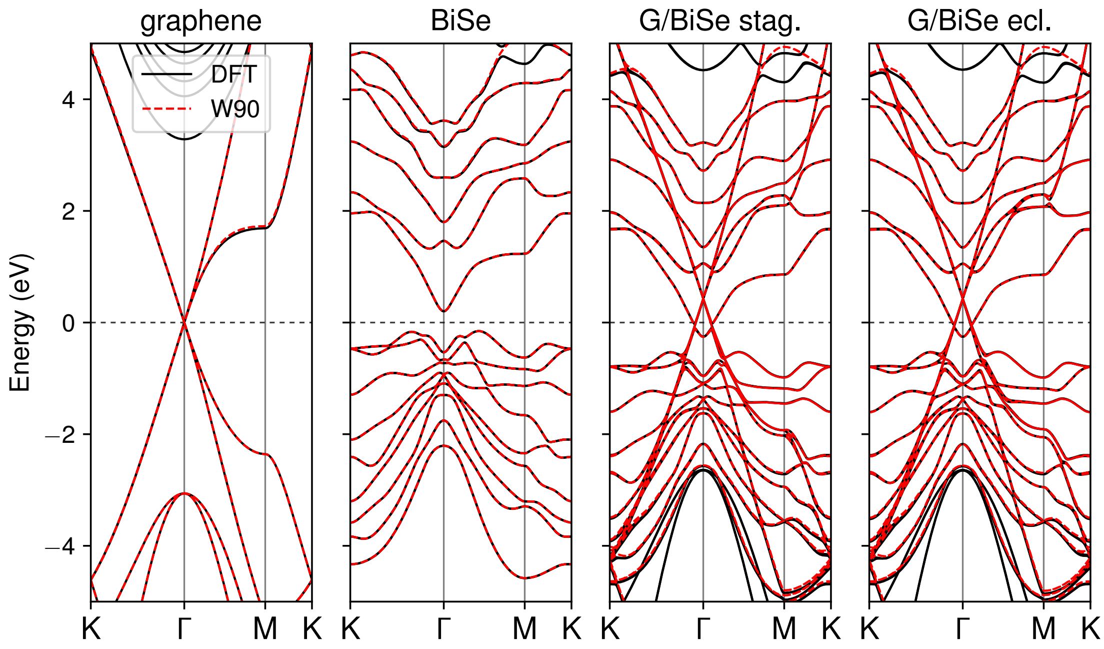

Figure S1: Comparison between the band structures calculated using density functional theory (DFT, black solid lines) and those obtained by Wannier interpolation (W90, red dashed lines) for graphene, Bi_2Se_3, and the G/Bi_2Se_3 heterostructure in staggered and eclipsed configurations. An excellent agreement is observed, particularly near the Fermi level (dashed horizontal line), confirming the accuracy of the Wannier representation in the energy range relevant for transport properties.

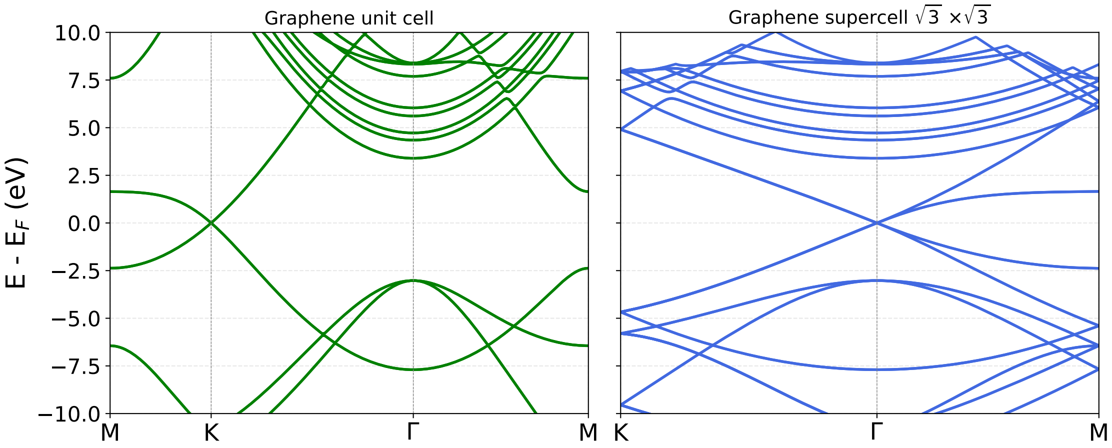

Figure S2: Electronic band structure of pristine graphene (left) and the $(\sqrt{3} \times \sqrt{3})R30^{\circ}$ supercell (right). Note the band folding of the Dirac cone from the K point to the Γ point in the supercell. The primitive lattice parameter was set to $a = 2.46$ Å.

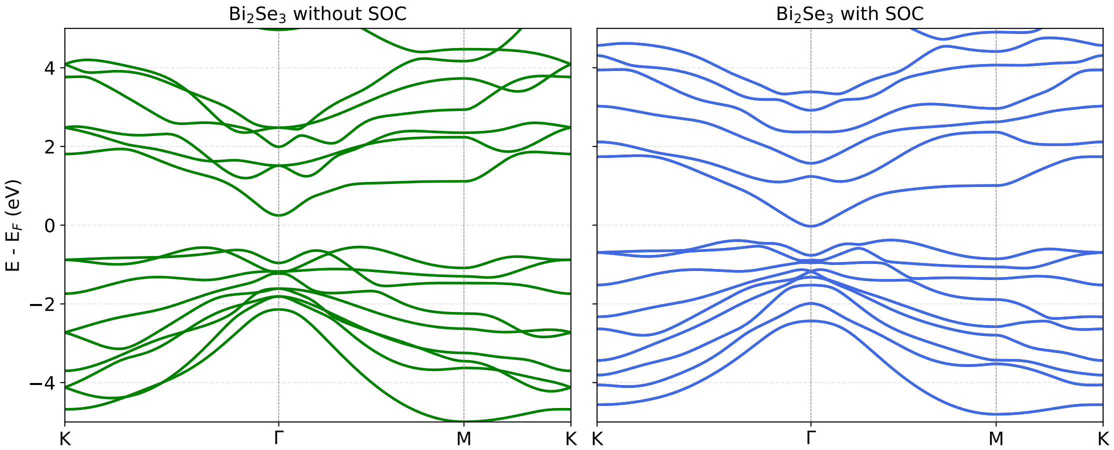

Figure S3: Electronic band structures without SOC (green), and with SOC (blue) of 1QL of Bi_2Se_3.

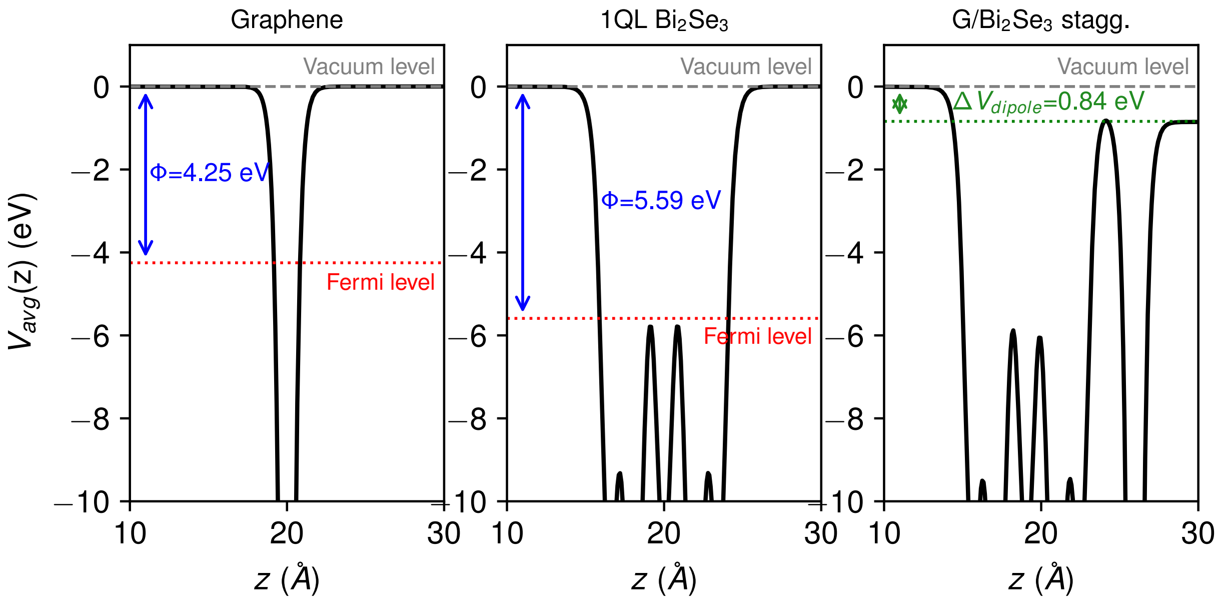

Figure S4: Planar-averaged electrostatic potential for graphene, 1QL of Bi_2Se_3, and G/Bi_2Se_3 heterostructure in the staggered configuration. The calculated work function value is shown in blue. For the heterostructure. We also indicate the interfacial dipole value in green color. The vacuum level is set to zero for both isolated graphene and Bi_2Se_3, whereas for the heterostructure the zero reference corresponds to the vacuum level at the Bi_2Se_3 side.

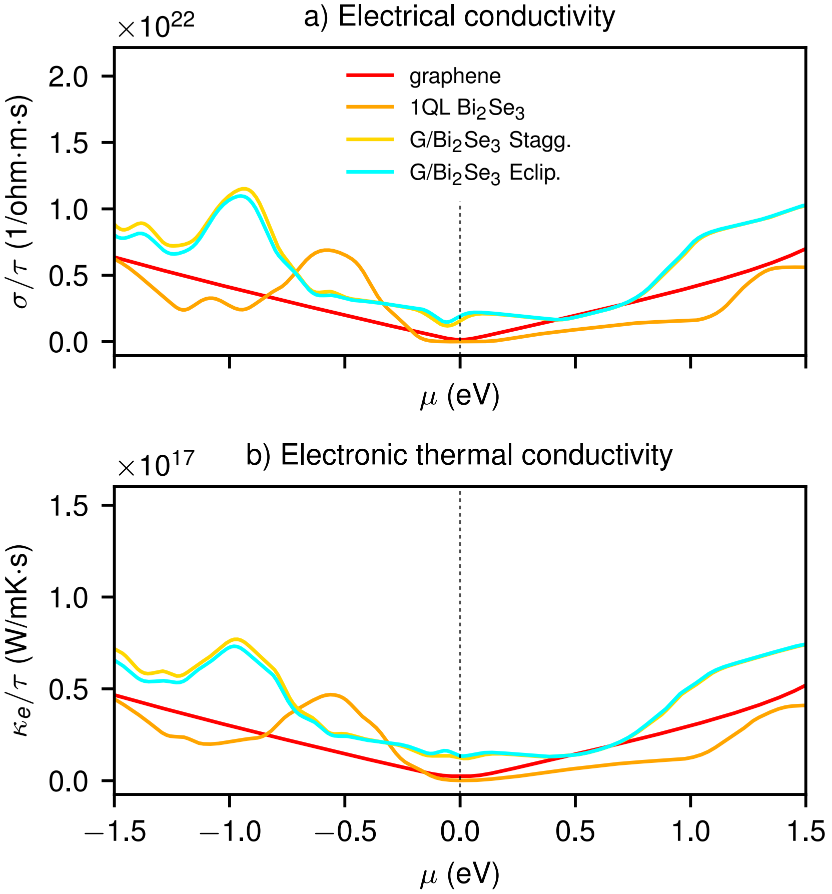

Figure S5: Comparison of transport properties as a function of the chemical potential (μ): (a) normalized electrical conductivity σ/τ and (b) electronic thermal conductivity κ_e/τ for graphene, a 1QL of Bi_2Se_3, and G/Bi_2Se_3 heterostructures in staggered and eclipsed configurations. The use of the ratios σ/τ and κ_e/τ eliminates the explicit dependence on the relaxation time τ, enabling a robust comparison across the different systems. The results show that the heterostructure exhibits enhanced electrical conductivity compared to the individual layers, providing clear numerical evidence of improved transport properties arising from interfacial effects.

As shown in Figure S6, the Seebeck coefficient of graphene is presented. In particular, the maximum values in the p-type region increase in agreement with the experimental reports summarized in Table S1. This consistency supports the validity of our approach based on Boltzmann transport theory within the constant relaxation time approximation (CRTA).

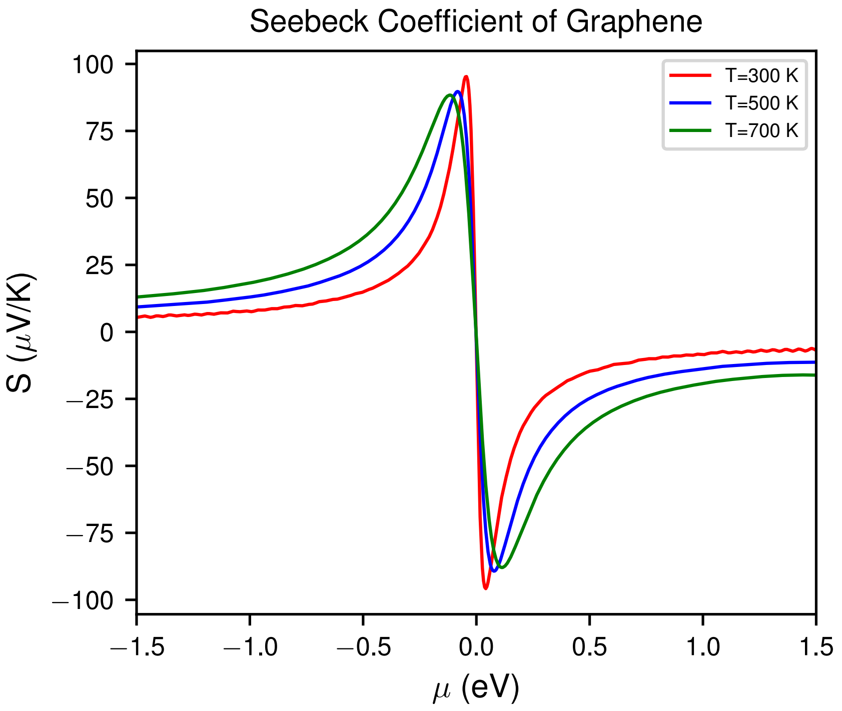

Figure S6: Seebeck coefficient of graphene as a function of chemical potential μ, calculated at $T = 300$, 500, and 700 K within the constant relaxation time approximation (CRTA). The maximum values in the p-type region are consistent with previously reported experimental and theoretical data (see Table S1), validating the accuracy and robustness of our Boltzmann transport implementation.

Table S1: Reported Seebeck coefficients for graphene from experimental and theoretical studies at $T = 300$ K. These values are included as reference benchmarks for comparison with the results obtained in this work.

Graphene (SLG)	Sebeeck	Reference
SLG/SiO_2	80×10^{-6}V/K	[Phys. Rev. Lett. 102 (9) 096807]
SLG/SiO_2	109×10^{-6}V/K	[Proc. Natl. Acad. Sci. U.S.A. 113 (50) 14272]
SLG/hBN	182×10^{-6}V/K	[Proc. Natl. Acad. Sci. U.S.A. 113 (50) 14272]
